# Supplementary material for: Dietary–Physical Activity Patterns in the Health Context of Older Polish Adults: The ‘ABC of Healthy Eating’ Project
Source: Nutrients. 2022 Sep 11;14(18):3757. doi: 10.3390/nu14183757 (PMC9506088; doi:10.3390/nu14183757)
Supplement: Supplementary file 1 [file nutrients-14-03757-s001.zip › nutrients-1881295-supplementary.pdf]

nutrients MDPI

Supplementary Materials

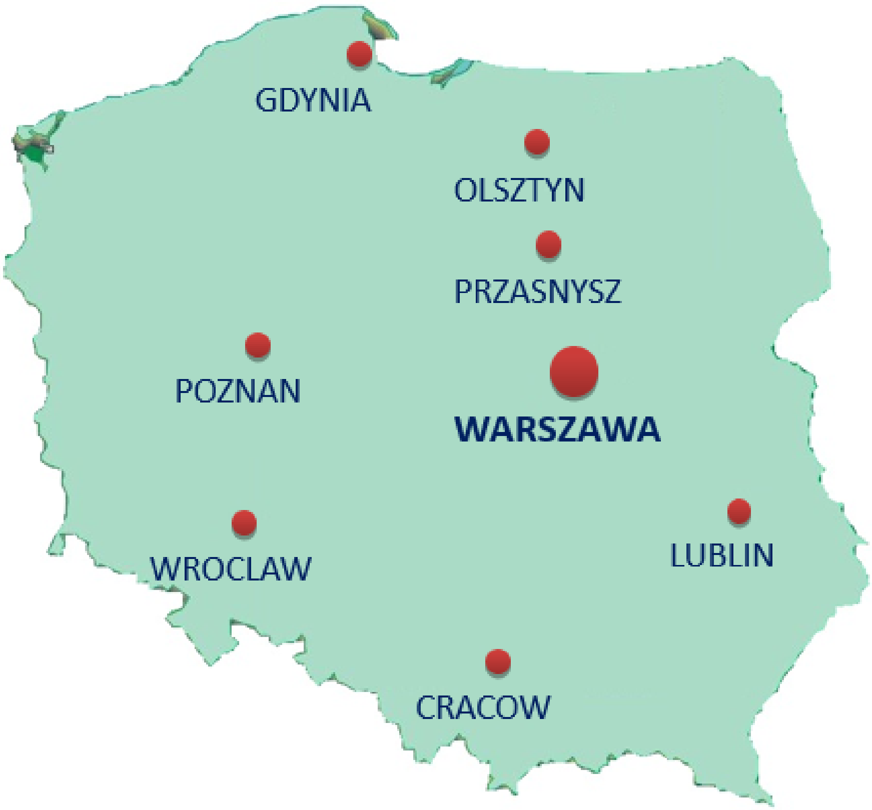

Figure S1. The location of academic centres involved in ABC of Healthy Eating study. Universities (cities) involved in the study: Warsaw University of Life Sciences, WULS-SGGW (Warsaw, Przasnysz); Gdynia Maritime University (Gdynia); University of Agriculture in Crakow (Cracow); University of Life Sciences in Lublin (Lublin); University of Life Sciences in Poznan (Poznan); University of Warmia and Mazury in Olsztyn (Olsztyn); Wroclaw University of Environmental and Life Sciences (Wroclaw).

nutrients

MDPI

Table S1. Characteristics of the PCA-derived Dietary-Physical Activity Patterns (D-PAPs) among Polish older adults (% of the sample).

Variables	Total sample	Dietary-physical activity patterns (tertiles)											
		'Pro-healthy eating and more-active'				'Sweets, fried foods, sweetened beverages'				'Juices, fish, sweetened beverages'			
		**bottom**	**middle**	**upper**	***p*-value**	**bottom**	**middle**	**upper**	***p*-value**	**bottom**	**middle**	**upper**	***p*-value**
Sample size (n)	361	121	120	120		121	120	120		120	120	121	
Food frequency consumption of:													
vegetables (times/day)[#]	0.93±0.59	0.50±0.38	0.89±0.44	1.41±0.54	<0.0001	1.04±0.65	0.87±0.52	0.88±0.59	ns	0.82±0.55	0.88±0.57	1.09±0.62	0.0006
never or almost never	1	2	1	0		0	2	1		2	0	1	
< 1 time/week	4	12	1	0		3	4	6		7	6	1	
1 time/week	5	14	2	0		4	3	8		5	4	7	
2-4 times/week	22	41	21	3	<0.0001	26	21	18	0.0354	25	27	13	0.0237
5-6 times/week	19	18	30	9		12	23	23		22	19	17	
1 time/day	30	10	36	44		28	35	27		27	28	35	
a few times/day	19	2	10	44		27	13	17		13	16	27	
fruits (times/day)[#]	0.97±0.64	0.48±0.40	0.92±0.49	1.53±0.53	<0.0001	0.92±0.67	1.03±0.60	0.98±0.66	ns	0.95±0.72	0.88±0.58	1.10±0.61	0.0103
never or almost never	3	7	2	0		5	0	4		8	1	1	
< 1 time/week	5	12	3	0		7	3	5		7	6	2	
1 time/week	6	13	3	1		6	3	8		7	7	3	
2-4 times/week	18	38	13	2	<0.0001	21	18	14	ns	18	22	14	0.0079
5-6 times/week	15	15	28	3		10	21	15		13	18	16	
1 time/day	30	12	38	40		30	31	30		23	32	36	
a few times/day	23	2	13	55		22	23	24		27	16	27	
water (times/day)[#]	1.53±0.69	1.04±0.78	1.64±0.58	1.90±0.31	<0.0001	1.45±0.73	1.53±0.68	1.60±0.65	ns	1.50±0.72	1.58±0.63	1.50±0.72	ns
never or almost never	3	9	0	0		6	3	1		5	0	4	
< 1 time/week	2	5	0	0		2	2	1		3	0	2	
1 time/week	2	7	1	0		1	3	4		1	2	5	
2-4 times/week	8	17	8	0	<0.0001	8	8	8	ns	7	10	7	ns
5-6 times/week	9	17	8	1		11	8	7		9	12	5	
1 time/day	11	9	13	9		12	11	9		11	9	12	
a few times/day	66	36	71	90		60	66	71		64	68	65	

dairy (times/day)[#]	0.78±0.57	0.50±0.46	0.75±0.50	1.09±0.57	<0.0001	0.81±0.62	0.76±0.53	0.76±0.55	ns	0.72±0.53	0.75±0.57	0.87±0.59	ns
never or almost never	3	7	2	1		3	3	3		3	0	6	
< 1 time/week	6	11	5	1		6	5	6		8	7	2	
1 time/week	10	17	8	3		9	10	10		8	13	8	
2-4 times/week	26	37	28	14	<0.0001	29	25	25	ns	30	31	18	0.0370
5-6 times/week	14	11	23	10		10	18	16		13	16	15	
1 time/day	29	12	26	48		26	30	29		29	22	35	
a few times/day	12	5	9	23		17	10	11		9	13	16	
grains (times/day)[#]	0.81±0.69	0.45±0.49	0.78±0.64	1.19±0.71	<0.0001	0.76±0.70	0.77±0.67	0.89±0.69	ns	0.83±0.72	0.81±0.69	0.77±0.66	ns
never or almost never	10	17	9	3		12	12	6		13	3	13	
< 1 time/week	6	12	4	3		7	5	8		8	8	3	
1 time/week	11	17	7	9		12	10	11		8	15	11	
2-4 times/week	21	25	31	8	<0.0001	23	25	16	ns	18	25	21	ns
5-6 times/week	11	12	15	5		10	12	10		9	10	12	
1 time/day	21	12	17	33		17	18	28		22	18	22	
a few times/day	20	5	18	38		20	18	23		23	21	17	
sweets (times/day)[#]	0.55±0.48	0.45±0.43	0.60±0.51	0.61±0.47	0.0087	0.20±0.20	0.45±0.29	1.00±0.47	<0.0001	0.62±0.51	0.49±0.49	0.54±0.43	ns
never or almost never	6	4	7	6		15	1	1		3	10	4	
< 1 time/week	12	17	10	10		29	8	0		15	12	11	
1 time/week	15	21	12	13		24	20	2		11	18	17	
2-4 times/week	31	33	33	28	ns	30	48	17	<0.0001	31	31	32	ns
5-6 times/week	7	7	8	6		1	10	11		8	7	7	
1 time/day	24	14	23	33		2	13	56		27	18	26	
a few times/day	5	3	7	4		0	0	14		7	5	2	
fried foods (times/day)[#]	0.28±0.25	0.27±0.22	0.31±0.26	0.26±0.25	ns	0.09±0.10	0.30±0.20	0.45±0.27	<0.0001	0.25±0.22	0.27±0.24	0.32±0.27	ns
never or almost never	9	6	9	11		25	0	1		10	7	9	
< 1 time/week	17	17	16	18		31	13	6		24	16	10	
1 time/week	30	36	23	32	ns	39	34	18	<0.0001	23	38	30	0.0480
2-4 times/week	37	37	41	33		5	50	57		38	33	40	
5-6 times/week	3	2	6	2		0	1	8		2	4	3	
1 time/day	4	3	5	5		0	3	11		3	3	7	

sweetened beverages (times/day)[#]	0.25±0.48	0.38±0.57	0.26±0.48	0.11±0.30	<0.0001	0.07±0.23	0.17±0.36	0.51±0.63	<0.0001	0.05±0.19	0.18±0.37	0.51±0.63	<0.0001
never or almost never	48	29	44	70		69	51	23		69	47	27	
< 1 time/week	20	21	25	14		19	21	20		22	27	12	
1 time/week	9	14	8	5		6	10	11		7	7	13	
2-4 times/week	9	16	6	4	<0.0001	2	9	14	<0.0001	2	9	15	<0.0001
5-6 times/week	3	4	4	1		2	2	6		0	3	7	
1 time/day	7	8	9	5		1	6	16		0	7	16	
a few times/day	4	8	4	1		1	2	11		1	2	11	
juices (times/day)[#]	0.51±0.54	0.38±0.43	0.59±0.55	0.55±0.62	0.0092	0.53±0.60	0.44±0.49	0.54±0.54	ns	0.10±0.15	0.43±0.34	0.98±0.59	<0.0001
never or almost never	14	17	8	17		19	13	9		33	8	1	
< 1 time/week	17	16	15	20		12	18	20		41	9	1	
1 time/week	14	18	13	12		14	18	10		16	20	7	
2-4 times/week	23	27	26	17	0.0002	21	23	25	ns	9	38	23	<0.0001
5-6 times/week	9	12	12	2		7	12	7		2	14	10	
1 time/day	16	7	19	23		16	11	23		0	11	38	
a few times/day	7	3	8	10		10	5	7		0	1	21	
fish (times/day)[#]	0.22±0.24	0.19±0.22	0.23±0.23	0.24±0.28	ns	0.24±0.29	0.19±0.19	0.23±0.24	ns	0.09±0.07	0.19±0.17	0.38±0.32	<0.0001
never or almost never	5	10	2	4		5	5	6		13	3	0	
< 1 time/week	27	29	28	25		26	28	28		43	33	5	
1 time/week	41	40	43	42		41	44	38		42	38	44	
2-4 times/week	20	16	23	23	ns	20	20	21	ns	2	24	35	<0.0001
5-6 times/week	2	2	2	2		2	0	3		0	1	5	
1 time/day	4	3	4	4		5	3	4		0	1	11	
a few times/day	0	0	0	1		1	0	0		0	0	1	
Physical activity													
practically no physical activity	2	4	2	2		3	3	2		2	3	3	
sedentary lifestyle	19	30	18	8		16	17	23		11	17	28	
light exercises at least 2-4 hours/week	40	41	44	33		39	45	35		35	46	38	
moderate intensive exercises for 1-2	20	15	22	24	<0.0001	15	19	27	0.0389	26	16	19	0.0105

hours/week or less intensive exercises												
>4 hours/week moderate intensive exercises	13	7	13	19		17	14	9		19	13	7
>3 hours/week intensive exercises regularly several times a week	6	2	2	13		11	3	4		8	6	4

Notes: #the frequency consumption was expressed as a times/day after assigning the values for categories of frequency consumption as follows: 'never or almost never'=0; '< 1 time/week'=0.06; '1 time/week'=0.14; '2-4 times/week'=0.43; '5-6 times/week'=0.79; '1 time/day'=1; 'a few times/day'=2; *p*-value – level of significance assessed by Chi^2 test (categorical variables) or Kruskal-Wallis' test (continuous variables); $p<0.05$; ns – statistically insignificant.
